# Identifying Challenges and Solutions for Improving Access to Mental Health Services for Rural Youth: Insights from Adult Community Members

**DOI:** 10.3390/ijerph21060725

**Published:** 2024-06-03

**Authors:** Janessa M. Graves, Demetrius A. Abshire, Elissa Koontz, Jessica L. Mackelprang

**Affiliations:** 1 WWAMI Rural Health Research Center, Department of Family Medicine, School of Medicine, University of Washington, Seattle, WA 98195, USA; 2 College of Nursing, Washington State University, Spokane, WA 99201, USA; elissa.koontz@wsu.edu; 3 College of Nursing, University of South Carolina, Columbia, SC 29208, USA; abshired@mailbox.sc.edu; 4 Department of Psychological Sciences, Swinburne University of Technology, Melbourne 3122, Australia; jmackelprang@swin.edu.au; 5 Global and Engagement, Federation University, Melbourne 3000, Australia

**Keywords:** rural, mental health, youth, equity, content analysis

## Abstract

In the rural United States, provider shortages, inadequate insurance coverage, high poverty rates, limited transportation, privacy concerns, and stigma make accessing mental healthcare difficult. Innovative, localized strategies are needed to overcome these barriers, but little is known about what strategies may be feasible in, or acceptable to, rural communities. We aimed to identify barriers youth face in accessing mental healthcare in rural Washington State and to generate ideas to improve access. Methods: Semi-structured, key informant interviews were conducted by telephone with adult community members, including parents, teachers, and healthcare providers. Participants answered questions related to barriers to mental healthcare access that confront youth and approaches to improving access. Detailed, de-identified field notes were analyzed using conventional content analysis. Results: Limited resources and stigma were the two primary barriers to accessing mental healthcare that youth encounter in the community. Limited resources included lack of services and transportation, inconsistent funding and mental health programming, and workforce shortages. Stigma associated with seeking mental healthcare was of particular concern for youth with diverse identities who experience additional stigma. Conclusions: Improving access to mental healthcare for rural youth will require building a strong mental health workforce and championing efforts to reduce stigma associated with help-seeking.

## 1. Introduction

The mental health of youth in the United States (US) is a substantial clinical and public health concern. Based on multiple federal data sources spanning 2013–2019, evidence suggests that approximately 20% of youth have experienced a major depressive episode, nearly 37% feel sad or hopeless, nearly 10% have ever experienced anxiety problems, and nearly 20% have seriously considered suicide [1]. During and since the COVID-19 pandemic, rising rates of psychological distress among youth have generated even greater concern in the US. For example, between 2019 and 2021, the prevalence of suicide ideation among female youth aged 14–18 years increased from 24% to 30% in the US [1,2,3]. In response to increasing concerns about youth mental health, the Office of the US Surgeon General issued an advisory in 2021 specifically focused on protecting youth mental health in the US [4].

There are also growing concerns about mental health in rural communities. For example, mental health and mental disorders were ranked as the fourth highest rural health priority among a national sample of rural stakeholders for 2010 [5] and 2020 [6] but were the “most oft-cited priority” for 2030 [7]. Scholars have emphasized that further research and innovative solutions are needed to address the mental health needs of rural communities [8].

Among youth, evidence suggests there is little variability in certain mental health outcomes across the rural–urban continuum [9]. For example, approximately 19% of children living in small rural areas have been diagnosed with a developmental, behavioral, or mental health disorder compared to approximately 15% in urban areas, 17% in large rural areas, and 16% in isolated areas [10]. Other evidence suggests that the prevalence of certain developmental conditions such as attention deficit hyperactivity disorder (ADHD), behavioral or conduct problems, depression, and anxiety is slightly higher among children in rural areas [1,11]. Death by suicide, however, is particularly pronounced among rural youth. Evidence suggests that rates of death by suicide are twice as high among rural compared to urban youth and that rural–urban disparities in death by suicide have been widening among boys [12]. These findings suggest there is a critical need to address more effectively the mental health of youth living in rural areas.

Although approximately 10% of both rural and urban youth receive treatment from a mental health provider [1], structural barriers impact access to mental health services in rural communities. These include, for example, a scarcity of mental health providers in rural communities. Compared to urban counties in the US, rural counties have 60% fewer psychologists, 33% fewer counselors, 29% fewer psychiatric nurse practitioners, and 40% fewer social workers per capita [13,14,15,16]. While the number of mental or behavioral health providers increased between 2014 and 2021, urban counties experienced a greater increase than rural areas [13,14,15,16]. Recently published research revealed that proportionally more rural older adults who received outpatient services for mood and anxiety disorders relied on generalist providers than urban-dwelling older adults. Those from rural areas also traveled twice as far for services as urban older adults [17]. Importantly, these disparities in access exist across the lifespan. For example, rural school districts report that lack of providers precludes comprehensive provision of mental health services in their schools [18], and rural counties have fewer youth mental health facilities than more urban areas [19]. The lack of mental health providers in rural communities is particularly alarming given that rural youth are more likely to die by suicide. In a study that examined the suicide deaths of 5034 young people aged 5 to 19 years who died in 2015 and 2016, youth suicide rates were 16% higher in counties designated as a mental health professional shortage area than those not [20].

The scarcity of mental health providers in rural communities requires rural dwellers to manage a considerable time and cost commitment to seek care compared to urban residents [17,21]. Longer travel times require that rural residents have access to reliable transportation, particularly given that public transportation may not be an option; a driver’s license; and the financial means for dedicated trips for healthcare services [22]. Rural residents may rely on social support networks to access to these resources, as prior research has suggested that rural residents have larger social networks and rely more on family members compared to urban residents [23].

High poverty rates in rural communities also contribute to healthcare access disparities; nationally, the poverty rate is 20% higher in rural areas compared to urban areas [24]. While telehealth may improve access to care in some rural communities, the absence of broadband internet and lack of computer technology compatible with telehealth hampers uptake of telehealth in many rural communities [25].

Privacy concerns related to seeking mental health services also impact rural residents, particularly rural youth who often rely on their parents to support their health needs. Specific privacy concerns include worries about stigma and potential social repercussions in small, close-knit communities where confidentiality may be difficult to maintain [26,27]. Misconceptions and negative attitudes around mental health issues are pervasive in some rural communities, which could contribute to lower likelihood of mental health service utilization among certain groups [27,28].

Given that there are multiple structural barriers to addressing mental health among rural youth, developing tailored, community-developed and -driven interventions may be one approach for improving youth mental health. Due to variability in how “rural” is defined and that resource availability varies across rural settings [29], localized efforts are needed to understand how specific rural communities might overcome barriers to addressing the mental health of rural youth. Thus, the aim of this study was to identify barriers to mental health services for youth from the perspective of adults in one rural Washington State community and to identify potential strategies that may be effective for overcoming these barriers. Finding from this study may be used to inform future approaches to better serving the mental health needs of youth in rural Northwest communities, where suicide rates are among the highest in the US [30,31,32].

## 2. Materials and Methods

This research was part of a larger community-based participatory research effort that sought to identify challenges to accessing youth mental health services and approaches to rectifying these challenges in three counties in rural eastern Washington State. This qualitative study involved semi-structured interviews with community stakeholders. Despite the potential for stigma about mental health to influence participation, we chose a qualitative approach to data collection, as we were interested in exploring in detail the concerns and lived experiences of rural-dwelling participants, as well as their ideas for enhancing mental health service delivery in their community.

### 2.1. Participants and Recruitment

Potential participants were identified from three rural counties in eastern Washington State. Participants were also asked to recommend others who may be interested in participating in this study (i.e., snowball sampling). All potential participants were identified as individuals involved with local youth in some way (i.e., parent, school personnel, healthcare provider). Potential participants received an email from the study coordinator describing the study and asking if they were interested in participating. If interested, they were contacted by telephone to discuss the study aims, to review the consent procedures, and to schedule an interview. Before conducting the interview, prospective participants were sent a consent document by email, which they signed and returned electronically.

### 2.2. Data Collection

Semi-structured interviews with key community informants were conducted in person or by telephone, based on participant preference. The interviews were led by a trained community member, who was also a member of the research team (EK). The research team collaboratively codesigned the 6-question interview guide with community members (Table 1). Questions included in the interview guide explored participants’ experiences engaging with youth mental health services from the perspective of a family member or caregiver, school personnel, or local healthcare provider.

In-person interviews were conducted in a private location selected by each participant. Telephone interviews were arranged at a time determined by each participant to ensure their comfort and privacy. Given well-documented concerns about privacy in small communities and the sensitive nature of the topic being explored in this study, the research team consulted with community partners (e.g., local health district and school leaders) at the project design stage and determined that the interviews would not be audio recorded. Instead, detailed field notes would be taken, as has been done in past studies [33]. During each interview and immediately thereafter, the interviewer (EK) recorded detailed field notes that captured participants responses to the interview prompts, including brief quotes, and contextual details. To maintain participant confidentiality, field notes for each participant interview were stored in a separate file that was assigned a unique number; participant identifiers were not included in those files. Each participant received a $20 Amazon.com gift card to thank them for participating in the study.

### 2.3. Analysis

Conventional content analysis [34] was used to characterize participants’ perspectives and to identify key categories related to youth access to mental health services in the rural community where the study was conducted. First, interview field notes were uploaded to Taguette (v.1.4.1-40), an online, open-source software tool for qualitative analysis [35]. Coding was conducted by two research team members (JMG and JLM), one of whom is a health services researcher and member of the rural community where the study was conducted, and the other is a rural-born clinical psychologist.

After familiarizing themselves with the field notes through reading and re-reading the files, the two researchers began to apply initial codes inductively. After several field note files had been coded, the two research team members met to discuss initial impressions of the data and to reflect on the emergent coding structure. As data analysis progressed, the coding structure was refined and codes that were redundant or that overlapped in conceptually meaningful ways were consolidated. Throughout this iterative process, code definitions were revisited and refined, codes were clustered and then grouped into key categories that represented the multidimensional facets of each of these categories. Potential relationships among key topic areas were then visually mapped.

### 2.4. Research Team Positionality

The research team comprised a health services academic with expertise in rural health research, a clinical psychologist, a nursing academic with expertise in rural health research, and a rural community member and parent who volunteers in a rural school. Two members of the research team (JMG and EK) reside in the rural community where the study was conducted. The other two (JLM and DAA) have spent substantial portions of their life living in rural US communities. Experiences of rural living informed research team members’ perspectives on the data, as did prior experiences of conducting participatory health-related research (JMG, JLM, and DAA) and delivering health and mental health services in rural communities and with marginalized populations (JLM and DAA).

## 3. Findings

**Figure 1:**
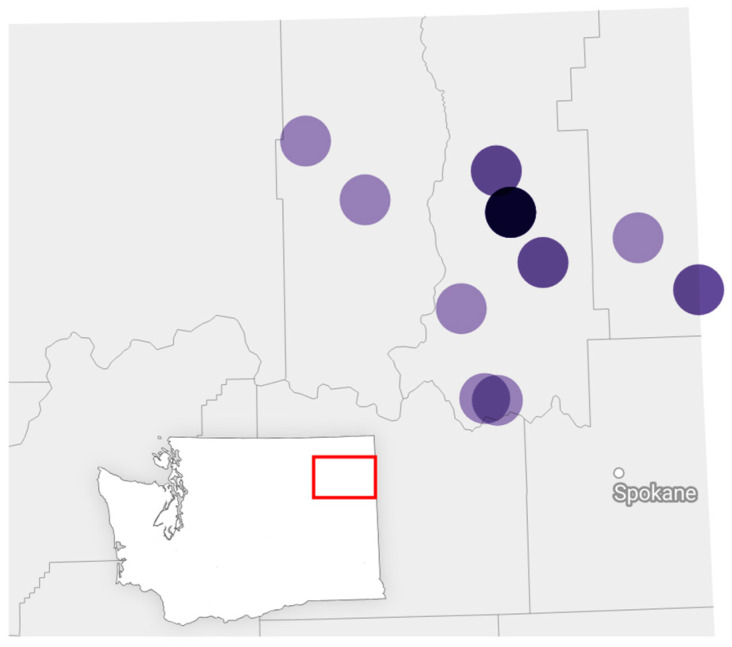
Geographic location of the study counties and approximate locations of participants (actual location adjusted slightly to protect participant confidentiality). Darker color circles indicate more participants. (Inset: Study location shown within map).

A total of 18 interviews were conducted between 5 January and 10 March 2021. Participants comprised four mental health providers (e.g., school counselor, therapist), eight parents of children under 18 years, 11 individuals who worked in schools (e.g., teacher, paraprofessional, program director), and four public health professionals from the Tri-County area (Figure 1).

**Figure 2:**
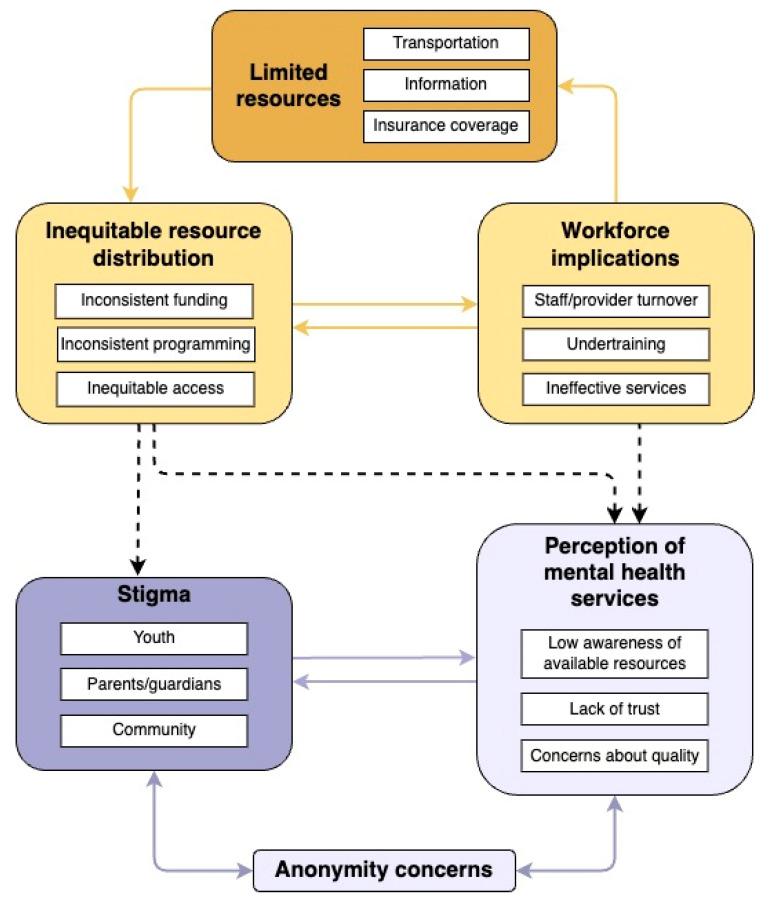
Conceptualized relationships between key categories discussed by community members; Limited resources (Category 1; gold) and Stigma (Category 2; purple).

Two key categories were generated from the field notes dataset: *limited resources* and *stigma*. While seemingly disparate topics, participants’ narratives captured the ways in which lack of resources and stigma are intertwined and linked by a web of coinciding factors that collectively contribute to the challenges that confront youth in the rural community who may benefit from mental health services (Figure 2). These barriers are explored in detail hereafter; potential participant-driven solutions are contextualized alongside existing research on rural health service delivery in the Discussion.

### 3.1. Limited Resources

Concerns related to limited mental health resources were ubiquitous among participants. However, limited resources extended beyond the sheer lack of mental health services in the local community. Limited resources included restricted hours for those services that do exist, few mental health providers in the community, and concerns about the qualifications of providers to support youth whose needs are particularly complex, including those who are at high risk of suicide. For instance, while school counselors were described as important supports available in some (but not all) schools, participants worried that some were not necessarily equipped to support young people in crisis situations.

Many participants also discussed transportation issues as a pervasive problem in the community, which placed logistical and financial burdens on families. For example, one participant described that parents must take time off work and drive long distances to access mental health services, effectively being forced to choose “between a paycheck and youth mental health”. In those situations, basic needs (e.g., food, housing) might be prioritized out of necessity. Public transportation was also a barrier to accessing mental health services. One participant raised the issue of a public bus service that had not yet resumed a year after the COVID-19 pandemic had been declared. Although telehealth was mentioned as a possible solution to overcoming transportation barriers, “unreliable internet” in some local areas represented yet another dimension of resource scarcity.

In addition to limited resources within the community, this topic also included resource scarcity experienced by individual families, including financial barriers to accessing mental health services. Lack of insurance coverage to fund mental healthcare was frequently mentioned as an issue for local families. Lack of information about existing mental health services was also cited as a concern, particularly given the role that parents often play as gatekeepers to mental healthcare for youth.

Alongside the barriers summarized above were two additional subtopics: *inequitable resource distribution* and *workforce implications*. Participants’ narratives suggested that these subtopics are both contributors to, and outcomes of, the persistent lack of resources experienced by the rural community. These subtopics are elaborated on hereafter.

#### 3.1.1. Inequitable Resource Distribution

Participants observed that mental health services were often inconsistently available in their community, which contributed to and/or exacerbated mental health inequities. For example, participants described frequent changes in the availability of services and the types of services offered, indicating that school-based mental health services are “hit or miss year to year”. Another interviewee in the same school district described how it was “really great” having a mental health therapist in the school “for a hot minute” but that provider is no longer there. Similar concerns about the high rate of staff turnover in Tribal mental health services were also mentioned.

There was also evidence of inequitable distribution of the resources that were present. For example, when speaking of the availability of mental health services in schools, one participant declared, “Some schools have, some don’t”, resulting in access disparities among youth. In those schools with mental health services, counsellors were sometimes available intermittently (e.g., “once every couple of weeks”), rather than continuously, which has implications for schools’ responsiveness to acute exacerbations of mental health difficulties, including suicide risk. This intermittency may also affect the therapeutic relationship and a youth’s long-term engagement with mental health services.

To overcome insufficient state funding for mental health services, one school leader described finding additional stopgap funding to provide services to youth, stating that they “hired [their] own mental health therapist… not paid by the prototypical funding model”. Inconsistency in funding and mental health programming has implications for securing qualified professionals to support the rural community and contributes to inequitable access to mental healthcare.

#### 3.1.2. Workforce Implications

Participants indicated that inconsistent funding and mental health programming in the community and local schools make it difficult for qualified providers to be recruited and retained, thus contributing to high staff turnover in services that need provider stability. One participant stated that the community had “been a revolving door of advocates and specialists”. This issue was also seen to contribute to the challenge of local providers being under-trained and ill-equipped to manage “big [mental health] situations”. Participants contended that an unreliable workforce contributed to some community members’ perceptions that mental health services for youth were ineffective. Another participant asserted that there is a pattern of youth seeking mental healthcare locally but “not feeling it’s successful”.

The combination of inconsistent (or non-existent) funding and workforce shortages was seen to contribute to a sense of fragmentation in services. Indeed, nearly all participants lamented having to “dig around” to find out where and how to access services and the ever-changing landscape of available services contributed to a sense of mistrust that perpetuated mental health stigma in the community.

### 3.2. Stigma

Concerns about stigma as a critical barrier to mental health help-seeking were voiced by all participants, as was the perception that stigma varies across generations and is less problematic among young people. Participants worried that even though the importance of tending to mental health was growing among youth, stigma among parents was a barrier to children and adolescents seeking mental health services. One participant indicated that “a lot” of parents suffer mental health issues themselves, but do not talk to anyone. As a result, “Kids see it as not normal to reach out”.

Aspects of intersectionality were also mentioned by participants. Some described instances of young people being minoritized due to their ethnoracial identity, specifically tribal populations and African American youth; sexual orientation; or gender identity. Participants raised concerns about lack of culturally safe care and worried that diverse identities are “not supported” within the community or, worse yet, be overtly excluded and subject to violence. Despite acknowledging that some existing services are progressive and open-minded, it was apparent that cultivating a safe, accepting environment where mental healthcare is normalized may feel like an uphill battle in the conservative community.

Participants spoke of the gatekeeping role that parents play in young people’s access to mental health services (e.g., driving them to appointments, paying for services). They explained that when parents do not view mental healthcare as a viable and valuable approach to supporting their child’s wellbeing, the challenge of overcoming resource-related barriers to mental healthcare in the community were compounded. Even for parents who might be keen for their child to receive mental health services, few options were available or stigma-related fears influenced the way such help might be sought. For instance, mental health stigma was seen to motivate community members to seek services elsewhere (e.g., travel to Spokane, 60 to 100 miles away), so that others would not know that they were accessing mental health services. Given the limited transportation options, it was apparent that this aspect of limited resources was likely fueling access disparities in the community.

Participants described the combination of mental health stigma and the experience that “everything is seen in a fishbowl” in the small community as a barrier to accessing existing mental health services locally. One participant asserted that maintaining anonymity is simply not possible when accessing local mental health clinics, stating, “You don’t want to park your car in front…people know you know what you drive.” For this reason, organizations such as the Boys and Girls Club were seen as underutilized options for mental health service delivery. In organizations that served a wider range of community members, they speculated that it would be easier to overcome fears about stigma and lack of anonymity that might otherwise be a deterrent. Similarly, the value of having services consistently available and normalized within schools was seen as essential.

#### Perception of Mental Health Services

Stigma both influenced and was influenced by resource-related barriers, including issues with workforce shortages and capabilities. Systemic factors related to limited resources and inequitable distribution of existing resources were seen by participants to exacerbate and further entrench the stigma associated with mental health help-seeking in the rural community, in part because inconsistent access to services and provider turnover contributed to “distrust” of mental health service providers, of the healthcare system, and of the value of mental health services more generally. For some participants, lack of trust was also discussed in relation to community members’ “skepticism” about the quality of the existing mental health services.

Participants pointed frequently to a lack of awareness of available mental health resources locally, suggesting that community members cannot rely on the services they might have known about in the past to still be available. Indeed, one participant commented that parents generally know their child is having a “problem”, but they “don’t know what to do”. This challenge was attributed partly to the fragmented and changeable local services and partly to gaps in knowledge about mental health more generally. Service turnover described by participants appears to make it difficult for community members to know what services they should turn to for what types of care, which thwarts trust-building and undermines confidence in the mental health system.

## 4. Discussion

The mental health of people living in rural communities has been recognized as one of the most pressing public health concerns for the past two decades [5,6,7]. Concerns regarding the mental health of rural youth are particularly alarming given the widening disparities in deaths by suicide among rural as compared to urban youth [1,2,3]. The multiple barriers to obtaining mental health services in rural communities exacerbate the issue. In this study, we sought to identify barriers to mental healthcare for youth in one rural Washington State community from the perspective of community-engaged adults and to identify potential strategies that may be effective for overcoming these barriers.

Traditional models of mental health treatment delivery whereby patients visit specialist providers are often not feasible for rural residents due to factors such as provider shortages and greater travel distances to locations where providers are available [36]. Instead, scholars have argued for alternative solutions in the rural context, such as community partnerships with schools, addressing less severe mental health issues in primary care settings, and increasing the number of parents and peers who are trained to address and respond to various mental health-related concerns [36]. However, one concern noted by participants in this study was the perception that people such as school counselors, when available, may lack the qualifications needed to address more severe mental health concerns. This finding underscores the importance of ensuring that existing supports receive proper training and continuing education to support rural youth, including those with acute needs.

Mental health providers who practice in rural settings or those in neighboring urban areas can serve as a valuable resource by training, monitoring, and consulting with others in the community who may be working directly with rural youth. This strategy, known as “task sharing” has been recommended by scholars as a strategy for improving mental health care delivery in rural and low resource areas [37].

Improving access to mental health services in rural areas requires close collaboration with rural community members to identify strategies that will be feasible and acceptable in their specific community context. Community engagement helps ensure that health policies and innovations are practical, localized, and culturally sensitive. The importance of working closely with rural communities to address mental health issues has been highlighted in existing literature. For example, an academic health center–community partnership was established to deliver telepsychiatry services in rural areas of Alabama in the US [38]. Lessons learned from this collaborative partnership included the importance of being flexible to meet the needs of both parties; the need to coordinate services for reimbursement purposes; and the potential need for lobbying efforts to reduce barriers, such as requiring a physician to be present at both the delivering and receiving sites.

Stigma was identified as an important obstacle to addressing the mental health of rural youth in the communities where the study was conducted. Prior research on rural–urban differences in stigma has highlighted that stigma is an important issue irrespective of geographic setting but may be particularly relevant for subgroups of rural residents. For example, Shroeder and colleagues found higher levels of stigma regarding mental illness among rural compared to urban women but no differences between rural and urban men [27]. Among a sample of older adults living in an isolated rural area, a rural area adjacent to a metropolitan area, and an urban area, researchers found that self-stigma and public stigma toward mental health services were highest among those living in isolated rural areas [39]. Moreover, older adults in the isolated rural community expressed a desire to address their psychological health privately. These sentiments echoed those articulated by participants in this study, who perceived stigma to be strongest among older generations in the community. Moreover, participants recognized that worries about not being able to seek mental health services anonymously in the small communities discourage mental health help-seeking. Among young gay men in Australia, those residing in rural areas were more likely to experience psychological distress, have lower self-esteem, and lower life satisfaction [40]. These findings are consistent with participants’ remarks in the current study that stigmatization surrounding the diversity of youth identities compounds the stigma associated with seeking mental health services.

Limited and inconsistently available resources in the rural communities in which this study was conducted appeared to exacerbate the mental health stigma already present in the geographically dispersed area. While participants suggested that more services were needed, two features appeared to be particularly important for mental health services: stability and location. Participants discussed the importance of services being consistent and reliable over time to build community trust and so people know where to turn in times of need. They also discussed increasing capability among providers in existing organizations that are already viewed favorably by the community, including schools, libraries, and community centers.

The US Surgeon General’s Advisory offers actionable steps to improve youth mental health, including steps that caregivers and family members, school personnel, and community organizations can take to address mental health challenges facing youth today [4]. Although the Surgeon General’s suggested interventions apply generally to US populations, they are strategies that can feasibly be employed in rural settings. For example, educators can be trained to recognize signs of changes in students’ mental, behavioral, or physical health. Family members and caregivers can promote youth mental health by role modeling positive behaviors, such as caring for their own mental health, which was discussed as a barrier among participants in this study. These actions would also help to normalize discussions about mental health, which is important for reducing stigma. In addition, family members and caregivers can help promote youth mental health by discussing the risks of substance use, being aware of warning signs, and monitoring online behaviors. Discussing risks associated with substance use is particularly important given evidence that youth in some rural areas are more likely than urban youth to use alcohol and certain illicit substances [41].

### Strengths and Limitations

A strength of this study was the qualitative design and use of conventional content analysis. This approach enabled the research team to center the lived experiences and perspectives of the research participants in the unique rural community wherein the study was conducted, rather than approaching data analysis with assumptions based on prior research [34].

Findings from this study should be interpreted in the context of at least three limitations. Given that rural youth may be less comfortable discussing issues regarding mental health with adults [42], this study focused on adult perspectives of the mental healthcare barriers facing rural youth. Rural youth may have different perspectives regarding barriers to accessing mental healthcare services, and future research is needed to understand youths’ perceptions of barriers. Second, findings from this study are based on detailed field notes rather than audio-recorded interviews. Although the decision to not audio record the interviews was based on community members’ input, recording the interviews would have almost certainly yielded more detailed insights into the challenges rural youth encounter in accessing mental healthcare services. However, we prioritized building trusting and respectful relationships with rural community members, as this is imperative for addressing health disparities. Our decision is consistent with other documented efforts to address mental health in which scholars have noted the importance of being flexible to meet community needs and preferences [38]. Finally, although a sample of 18 is appropriate in qualitative research of this nature, there may be limits to the transferability of our findings to other contexts and geographic areas. Similarly, we appreciate that each rural community is unique and some of the barriers described by study participants may or may not be transferable to other rural communities in the US or internationally.

## 5. Conclusions

Mental health is a leading health concern facing rural communities. In this study, adults in rural Pacific Northwest US communities discussed limited resources and stigma as two of the most significant barriers to addressing the mental health of local youth. To better address the mental health needs of rural youth in the community, the number of trained mental health providers must be increased, the capabilities of school-based mental health supports must be expanded, and efforts to reduce stigma must be prioritized. Policies that ensure the stability of services from a funding standpoint and that ensure that youth and families can access those services (e.g., transportation options, expanded insurance coverage for mental healthcare services) are crucial to optimizing access to mental health services among rural youth.

## Figures and Tables

**Table 1:** Semi-structured interview guide.

1. Tell us about your relationship to this community.
2. From your experience, can you tell us a little about the types of mental health resources and services that are available to children and adolescents in this community?
3. From your experience, what challenges might children and adolescents face in accessing mental health services in this area?
4. From your experience, what barriers might children and adolescents face in accessing mental health services in this community?
5. From your experience, in what ways might we better serve children and adolescents to access mental health services in this community?
6. Our goal today was learn about your experiences related to child and adolescent mental health services in this community. You have all shared a lot of valuable information. Is there anything else you want to say that you didn’t get a chance to say yet? Is there anything we missed?

## Data Availability

The data are not publicly available due to their containing information that could compromise the privacy of research participants or their families.
